# Intranasal adenovirus-vectored Omicron vaccine induced nasal immunoglobulin A has superior neutralizing potency than serum antibodies

**DOI:** 10.1038/s41392-024-01906-0

**Published:** 2024-07-22

**Authors:** Si Chen, Zhengyuan Zhang, Qian Wang, Qi Yang, Li Yin, Lishan Ning, Zhilong Chen, Jielin Tang, Weiqi Deng, Ping He, Hengchun Li, Linjing Shi, Yijun Deng, Zijian Liu, Hemeng Bu, Yaohui Zhu, Wenming Liu, Linbing Qu, Liqiang Feng, Xiaoli Xiong, Baoqing Sun, Nanshan Zhong, Feng Li, Pingchao Li, Xinwen Chen, Ling Chen

**Affiliations:** 1 Guangzhou Institute of Infectious Disease, Guangzhou Eighth People’s Hospital, Guangzhou Medical University, Guangzhou, China; 2 Guangzhou National Laboratory, Guangzhou, China; 3 State Key Laboratory of Respiratory Disease, Center for Cell Lineage Research, Guangzhou Institutes of Biomedicine and Health, Chinese Academy of Sciences, Guangzhou, China; 4 University of Chinese Academy of Sciences, Beijing, China; 5 State Key Laboratory of Respiratory Disease, National Clinical Research Center for Respiratory Disease, Guangzhou Institute of Respiratory Health, the First Affiliated Hospital of Guangzhou Medical University, Guangzhou, China; 6 Xiamen United Institute of Respiratory Health, Xiamen, China

**Keywords:** Immunology, Infectious diseases

## Abstract

The upper respiratory tract is the initial site of SARS-CoV-2 infection. Nasal spike-specific secretory immunoglobulin A (sIgA) correlates with protection against Omicron breakthrough infection. We report that intranasal vaccination using human adenovirus serotype 5 (Ad5) vectored Omicron spike in people who previously vaccinated with ancestral vaccine could induce robust neutralizing sIgA in the nasal passage. Nasal sIgA was predominantly present in dimeric and multimeric forms and accounted for nearly 40% of total proteins in nasal mucosal lining fluids (NMLFs). A low-level IgG could also be detected in NMLFs but not IgM, IgD, and IgE. After a complete nasal wash, sIgA in the nasal passage could be replenished rapidly within a few hours. A comparison of purified paired serum IgA, serum IgG, and nasal sIgA from the same individuals showed that sIgA was up to 3-logs more potent than serum antibodies in binding to spikes and in neutralizing Omicron subvariants. Serum IgG and IgA failed to neutralize XBB and BA.2.86, while nasal sIgA retained potent neutralization against these newly emerged variants. Further analysis showed that sIgA was more effective than IgG or IgA in blocking spike-mediated cell-to-cell transmission and protecting hACE2 mice from XBB challenge. Using a sIgA monoclonal antibody as a reference, we estimated that the total nasal sIgA contains about 2.6–3.9% spike-specific sIgA in NMLFs collected approximately one month after intranasal vaccination. Our study provided insights for developing intranasal vaccines that can induce sIgA to build an effective and mutation-resistant first-line immune barrier against constantly emerging variants.

## Introduction

Severe acute respiratory syndrome coronavirus 2 (SARS-CoV-2) is the causative agent of the coronavirus disease 2019 (COVID-19) pandemic that has claimed the lives of more than 7.05 million people worldwide since the outbreak in 2019.^1^ SARS-CoV-2 initially infects epithelial cells in the nasopharynx by using the receptor-binding domain (RBD) of spike protein to interact with the angiotensin-converting enzyme 2 (ACE2) receptor. Since the end of 2021, Omicron subvariants have become the dominant circulating strains, capable of immune evasion and rapid transmission. Omicron subvariants preferentially infect the upper respiratory tract, particularly the nasal passage. Intramuscularly administered vaccines can reduce COVID-19 disease severity and mortality, but are ineffective in blocking infection with Omicron variants that first infect and replicate in the upper airway mucosa, especially in the nasal cavity. It is reported that one month after the fourth dose of mRNA vaccine, vaccine efficacy against symptomatic Omicron infection was 11–30%. Most of these Omicron infected individuals have a high viral load in the nasopharyngeal tract and can therefore be highly contagious.^2,3^

People who had multiple doses of vaccination plus a previous infection developed the so-called “hybrid immunity”, and were best protected against future symptomatic Omicron infection than people who only received mRNA vaccines.^2^ Anti-RBD IgA in saliva with neutralizing activities can be detected in SARS-CoV-2-infected patients.^4^ Spike-specific mucosal sIgA can be detected in the nasal swabs of individuals who recovered from a SARS-CoV-2 infection.^5^ After recovery from a previous SARS-CoV-2 infection, people with a lower level of SARS-CoV-2-specific sIgA in the nasal passage have a higher risk of reinfection.^6^ The higher level of spike-specific sIgA, but not spike-specific IgG in the nasal fluid or saliva correlated with better protection against Omicron breakthrough infections.^7,8^ A recent report revealed that SARS-CoV-2-specific sIgA in the nasal passage wanes 9 months after infection and could not be induced by subsequent intramuscular vaccination.^5^ Therefore, the induction and persistence of mucosal spike-specific sIgA by an intranasal vaccine may provide a better protection against infection. Unlike serum IgA, which is predominantly present as a monomer (mIgA), mucosal sIgA is produced by plasma cells located in the lamina propria below the epithelium and secreted mainly as a dimer linked by a joining (J) chain (dIgA). dIgA binds to the polymeric immunoglobulin receptor (pIgR) on the basolateral side of mucosal epithelial cells, is transported to the apical side with the addition of a secretory component (SC) from pIgR, and then is released into the lumen.^9^ IgA has two subclasses, IgA1 and IgA2, with IgA1 constituting more than 90% of IgA in the upper respiratory tract.^4^^,10^ The hinge region of IgA1 contains 26 amino acids with glycosylation, whereas the hinge region of IgA2 contains 13 amino acids and lacks glycosylation. sIgA is critical in protecting respiratory mucosa by neutralizing viruses and impeding their attachment to epithelial cells.^11^ In contrast, intramuscularly injected vaccines induced circulating IgG but not mucosal sIgA. Therefore, intranasal vaccination to mimic viral infection in the upper respiratory tract may establish a better mucosal immune barrier to block infection.

Ad5, as a non-disease-associated respiratory virus, deletion of E1 region renders the virus replication incompetent, and so it can serve as vectors for a variety of infectious diseases to deliver vaccine antigens of interest. Earlier clinical trials have shown that Ad5-based vaccines via intranasal administration is well tolerated and safe.^12,13^ We reported in 2020 that intranasal vaccination using a replication-incompetent Ad5 expressing spike could elicit sterilizing-like protection against SARS-CoV-2 challenge in rhesus macaques.^14^ The pharyngeal viral loads on day 10 after the challenge were undetectable, and there was no elevation of serum-neutralizing antibodies after the challenge, demonstrating that the instilled viruses were eliminated without further proliferation to boost the immune response.^14^ We later generated Ad5-S-Omicron BA.1, an Ad5 expressing Omicron BA.1 spike (NB2155), and demonstrated that intranasal vaccination in mice primed with injected vaccine could elicit respiratory mucosal sIgA and T cell immune responses against both pre-Omicron strains and Omicron variants.^15^ Importantly, nasal mucosal lining fluids (NMLFs) collected from an intranasally vaccinated person possessed broadly neutralizing activities and could protect hACE2 mice against Omicron BA.1 challenge upon intranasal instillation.^15^ In a clinical study using this vaccine, we observed that people who completed a two-dose intranasal vaccination regimen were well protected from infection during the Omicron BA.5 outbreak when China lifted the zero-COVID policy in December 2022 (manuscript in revision). However, the roles of nasal mucosal antibodies and the underlying mechanisms in preventing SARS-CoV-2 infection need to be further understood.

In the present study, we purified paired serum IgA, nasal sIgA, and serum IgG collected simultaneously from the same individuals, and compared their neutralizing potency, breadth, and spike-binding activity. We compared their abilities in blocking spike-mediated syncytia formation and protecting hACE2 mice from XBB challenge. In addition, we assessed the amount and concentration of spike-specific sIgA and total sIgA in the nasal passage in adults.

## Results

### Intranasal vaccination induces high molecular weight nasal mucosal sIgA with broadly neutralizing activities against all tested variants

**Fig. 1:**
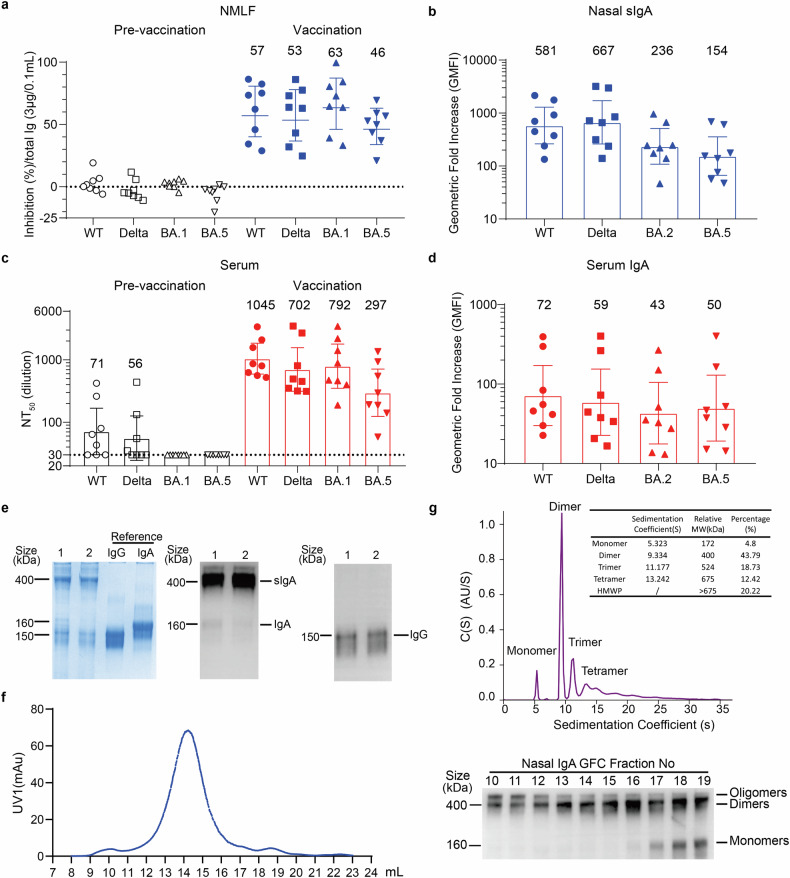
Analysis of immunoglobulins in nasal mucosal lining fluids (NMLFs). **a** Neutralizing activities of NMLFs against VSV pseudoviruses bearing spikes of Wildtype (WT), Delta, Omicron BA.1, and BA.5. NMLFs were collected from eight donors before and 3–6 weeks after two intranasal booster doses. Neutralization assay was performed using 100 µl samples containing an equal amount of total IgA (3 μg) and presented as the percentage of inhibition. Data are shown as % inhibition. Individual data are presented (*n* = 8). **b** Detection of IgA that binds to spikes of WT, Delta, BA.2, and BA.5 in NMLFs. NMLFs from eight donors were collected before and 3–6 weeks after two intranasal doses. Spike-specific IgA was detected using an electrochemiluminescence method (Meso Scale Discovery). Individual data were normalized to equal amounts of total protein. Data are shown as the geometric mean fold increase (GMFI) with a 95% confidence interval (CI). Individual data are presented (*n* = 8). **c** Neutralizing activities of serum samples against VSV pseudoviruses bearing spikes of WT, Delta, BA.1, and BA.5. Serum samples were collected from eight donors before and 3–6 weeks after intranasal vaccination. A neutralization assay was performed using serial dilutions of the samples, and the results are presented as neutralizing titers (NT_50_). The cutoff value was set at 1:30. Data are shown as the geometric mean titer (GMT) with 95% CI. Individual data are presented (*n* = 8). **d** Detection of IgA that binds to the spikes of WT, Delta, BA.2, and BA.5 in serum samples. Serum samples from eight donors were collected before and 3–6 weeks after two intranasal booster doses. Spike-specific IgA was detected using an electrochemiluminescence method (Meso Scale Discovery). Data are shown as GMFI with 95% CI. Individual data are presented (*n* = 8). **e** Sodium dodecyl sulfate-polyacrylamide gel electrophoresis (SDS‒PAGE) and western blot analysis of NMLFs. 8 μg total protein in NMLFs of two representative donors were loaded onto an SDS-PAGE gel and stained with Coomassie blue. Purified human serum IgG and IgA were used as references. Western blot analysis was performed with 200 ng total protein in NMLFs, using an anti-human IgA heavy chain (HC) antibody and an anti-human IgG H + L antibody to detect IgA and IgG, respectively. **f** Nasal sIgA samples were purified and pooled from NMLFs of six donors, then subjected to gel-filtration chromatography (GFC) separation. Each collected fraction was subjected to western blot analysis using an anti-human IgA heavy chain (HC) antibody. **g** Molecular size distribution of nasal sIgA. Nasal sIgA were purified and pooled from NMLFs of six donors, then subjected to sedimentation velocity-analytical ultracentrifugation (SV-AUC). The data were analyzed with SEDFIT software to obtain sedimentation coefficient distribution C (S)

To evaluate and compare the nasal mucosal and systemic antibody responses induced by intranasal vaccination, we collected nasal lavage and serum samples from 8 elite donors at 3–6 weeks after two doses of intranasal spray of NB2155 (Ad5-S-Omicron BA.1). All donors have received 2 or 3 inactivated whole-virus vaccine via intramuscular injection at least six months ago and have never been infected with SARS-CoV-2 during the study period (Supplementary Table 1). After intranasal vaccination, neutralizing antibody activities against BA.1, BA.5, Wildtype (WT), and Delta could be detected in concentrated NMLFs (Fig. 1a). Using an electro-chemiluminescent method, spike-specific sIgA in NMLFs showed a significant geometric mean fold increase (GMFI) against the spikes of WT (581-fold), Delta (667-fold), BA.2 (236-fold), and BA.5 (154-fold) than before intranasal vaccination (Fig. 1b). There was a significant increase but to a less extend of serum spike-specific IgA against WT (72-fold), Delta (59-fold), BA.2 (43-fold), and BA.5 (50-fold) than that before intranasal vaccination (Fig. 1d). Serum neutralizing geometric mean titer (GMT) against pseudovirus WT and Delta also increased from 1:71 and 1:56 before intranasal vaccination to 1:1045 and 1:702, while GMT against BA.1 and BA.5 increased from below 1:30 before intranasal vaccination to 1:792 and 1:297 (Fig. 1c).

**Fig. 2:**
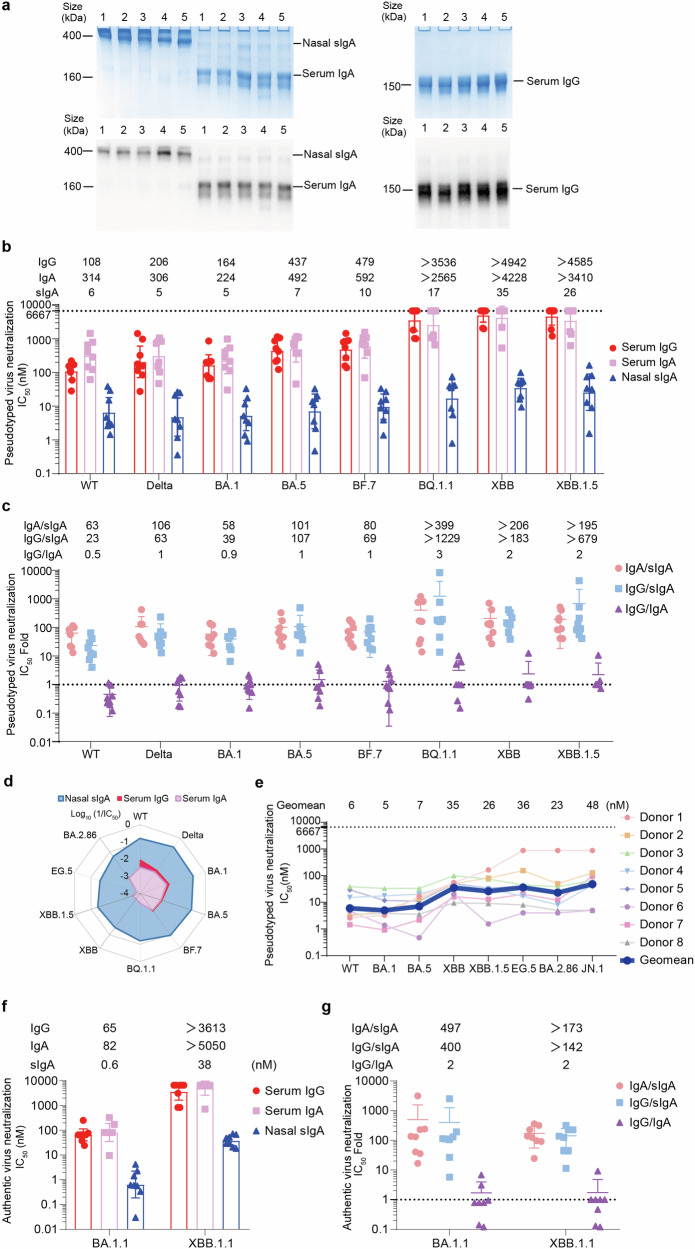
Neutralizing activities of paired nasal sIgA, serum IgG, and serum IgA from the same individuals. **a** Paired nasal sIgA, serum IgG, and serum IgA were simultaneously purified from the same individuals. Each sample was evaluated using SDS-PAGE and Coomassie blue staining. Western blot analysis was performed using an anti-human IgA heavy chain (HC) antibody and an anti-human IgG H + L antibody to detect IgA and IgG. Representative data from 5 donors are shown. **b** Neutralizing activities of eight paired purified nasal sIgA, serum IgG, and IgA samples against VSV pseudoviruses bearing spikes of pre-Omicron WT and Delta strains, as well as Omicron subvariants BA.1, BA.5, BF.7, BQ.1.1, XBB, XBB.1.5. A neutralization assay was performed using serial dilution of samples, and the results are presented as on the geometric mean 50% inhibitory concentration (IC_50_) in nM. The molecular weight of dimeric sIgA was used to convert the unit between mass and molar concentrations. The formula for converting mass concentration to molar concentration is Molar concentration (M) = Mass (mg)/(Volume (mL) × Molecular weight (g/mol or Da)). Namely, nM = μg/mL ÷ Molecular weight (Da) × 10^6^. For serum IgG and serum IgA, we used molecular weights of 150 kDa and 160 kDa for the conversion between mass concentration and molar concentration, respectively. For calculation purposes, samples without detectable neutralizing activity at the highest concentration (1000 μg/mL) were assigned an IC_50_ value of 6666.7 nM as no neutralization (dashed line). Data are shown as the geometric mean IC_50_ with 95% CI. Individual data are presented (*n* = 8). **c** The ratios of IC_50_ between paired IgA/sIgA, IgG/sIgA, and IgG/IgA for each donor are presented. The ratio of IgA IC_50_ to sIgA IC_50_ was firstly calculated for each donor, then calculated the average ratio of eight donors as IgA/sIgA. Similarly, the ratio of IgG IC_50_ to sIgA IC_50_ was first calculated for each donor, then calculated the average ratio of eight donors as IgG/sIgA. The data shown are the mean values of all individual paired ratios (mean ± SD, *n* = 8). **d** The neutralization spectra of nasal sIgA, serum IgG, and serum IgA. The radar charts were drawn based on the geometric mean IC_50_ titers of purified nasal sIgA, serum IgG, and serum IgA against pseudoviruses bearing spikes of Omicron subvariants BA.1, BA.5, BF.7, BQ.1.1, XBB, XBB.1.5, EG.5, BA.2.86, and pre-Omicron WT and Delta. **e** Neutralizing activities of purified nasal sIgA against VSV pseudoviruses bearing spikes of WT, BA.1, BA.5, XBB, XBB.1.5, EG.5, BA.2.86, and JN.1. A neutralization assay was performed using serial dilutions of samples, and the results are presented as 50% inhibitory concentration (IC_50_) in nM. Data are shown as geometric mean half-maximal inhibitory concentration (IC_50_) with 95% CI. Individual data are presented (*n* = 8). **f** Neutralizing activity of 8 paired purified nasal sIgA, serum IgG, and serum IgA samples against authentic viruses BA.1.1 and XBB.1.1. The neutralization assay was performed using serial dilutions of samples, and the results are presented as the geometric mean IC_50_ values in nM. For calculation purposes, samples without detectable neutralizing activity at the highest concentration (1000 µg/mL) were assigned an IC_50_ value of 6666.7 nM as no neutralization (dashed line). Data are shown as geometric mean IC_50_ with 95% CI. Individual data are presented (*n* = 8). **g** The ratios of IC_50_ between paired IgA/sIgA, IgG/sIgA, and IgG/IgA for each donor are presented. Data shown are the mean values of all individually paired ratios (mean ± SD, *n* = 8)

To assess the composition of immunoglobulin isotypes in NMLFs, NMLFs were first analyzed using sodium dodecyl sulfate-polyacrylamide gel electrophoresis (SDS-PAGE) and western blot (Fig. 1e). Nasal sIgA appeared mostly as dimeric and multimeric forms with molecular weights around 400 kDa or higher on western blot (Fig. 2a). A very low level of IgG was detected in NMLFs with molecular weights of approximately 150 kDa (Fig. 1e). No IgM, IgD, and IgE were detected by western blot using respective anti-IgM, anti-IgD, and anti-IgE antibodies (Supplementary Fig. 2). Further analysis of purified nasal sIgA pooled from 6 donors using size-exclusion chromatography and sedimentation velocity-analytical ultracentrifugation demonstrated that nasal sIgA consist of monomers (4.8%), dimers (43.8%), trimers (18.7%), tetramers (12.4%), and higher molecular weight oligomers (20.2%) (Fig. 1f, g).

### Nasal sIgA has superior neutralizing potency and spike-binding activity than serum IgA and IgG

To compare the neutralizing potency of nasal sIgA with that of serum antibodies, we first purified serum IgG, serum IgA, and nasal sIgA from each person. Each individual’s samples were collected simultaneously to ensure a paired comparison of serum antibodies and nasal sIgA. Purified serum IgA and IgG were monomeric, with molecular weights of approximately 160 and 150 kDa. Purified nasal sIgA appeared mostly as dimeric and multimeric forms with molecular weights around 400 kDa or higher on western blot (Fig. 2a). Neutralizing activities of paired serum IgA, serum IgG, and nasal sIgA were measured by using a panel of pseudoviruses. Nasal sIgA had a 50% inhibitory concentration (IC_50_) of 5, 7, and 10 nM GMT against BA.1, BA.5, and BF.7 and was 39-, 107-, and 69-fold stronger than IgG, which had an IC_50_ of 164, 437, and 479 nM GMT against these Omicron early subvariants, respectively. sIgA had IC_50_ of 6 and 5 nM GMT in neutralizing WT and Delta, which were 23- and 63-fold stronger than IgG in neutralizing WT and Delta. IgA showed comparable neutralizing potency as IgG, with an IgG/IgA neutralizing ratio near 1.0 for most strains with detectable IgG and IgA neutralizing activities (Figs. 2b, c, Supplementary Table 2, Supplementary Table 3). IgG and IgA from most donors nearly lost their neutralizing activity against late Omicron variants XBB, XBB.1.5, and BQ.1.1. sIgA had an IC_50_ of 35, 26, and 17 nM GMT against XBB, XBB.1.5, and BQ.1.1, respectively, which were 2–3 logs more potent than serum IgG and IgA (Fig. 2b, c, Supplementary Table 2, Supplementary Table 3). During the submission and revision of this manuscript, the Omicron subvariant EG.5, BA.2.86, and JN.1 sequentially became the major circulating strains. We also evaluated the neutralizing potency of sIgA using pseudoviruses bearing EG.5, BA.2.86, and JN.1 spikes. sIgA had an IC_50_ of 36 nM, 23 nM, and 48 nM against EG.5, BA.2.86, and JN.1, which is comparable to the IC_50_ for XBB (35 nM), while IgG and IgA had no neutralization (Fig. 2e, Supplementary Table 2, Supplementary Table 3). Although this intranasal vaccine used BA.1 spike as the antigen, the sIgA neutralizing spectra on the radar plot appeared as a well-rounded umbrella against 10 strains. In contrast, the IgG and IgA neutralizing spectra on the radar plot appeared as a triangle due to no neutralization against Omicron subvariants beyond BQ.1.1 (Fig. 2d). This finding demonstrated that nasal mucosal sIgA is more resistant to antibody evasion by emerging variants than serum IgG and IgA, indicating that the intranasal vaccine can potentially be variants-proof.

We verified the results of pseudovirus neutralization using authentic BA.1.1 and XBB.1.1 viruses by a plaque-reduction neutralization test (PRNT). Consistent with pseudovirus neutralization, sIgA showed IC_50_ of 38 nM GMT against authentic XBB.1.1. In contrast, serum antibodies (IgA and IgG) from most individuals had no or extremely weak neutralizing activity at the maximum test concentration (1000 μg/mL) (Fig. 2f). sIgA showed IC_50_ of 0.6 nM GMT against authentic BA.1.1, which was 497-fold and 400-fold stronger than serum IgA (IC_50_ = 82 nM) and IgG (IC_50_ = 65 nM), respectively (Fig. 2f, g).

Eight SARS-CoV-2 spike proteins specific-binding antibody activities of IgA, IgG, and sIgA were also measured by enzyme-linked immunosorbent assay (ELISA). sIgA possessed greater binding activities than IgG for the pre-Omicron strains WT (18-fold) and Delta (11-fold), as well as for BA.1 (54-fold), BA.2.12.1 (23-fold), BA.5 (20-fold), BF.7 (25-fold), BQ.1.1 (18-fold), and XBB (18-fold) (Supplementary Fig. 1a, b). sIgA also showed greater binding activities than IgA for BA.1 (130-fold), BA.2.12.1 (49-fold), BA.5 (42-fold), BF.7 (41-fold), BQ.1.1 (54-fold), and XBB (62-fold), as well as WT (60-fold) and Delta (37-fold) (Supplementary Fig. 1a, b). The binding activity of IgA to spike proteins was approximately 50% lower than that of IgG (Supplementary Fig. 1a, b). Therefore, nasal sIgA is stronger than serum IgA and IgG in binding to spike protein of both pre-Omicron and Omicron subvariants.

### Nasal sIgA is superior to serum IgA and IgG in inhibiting spike-mediated syncytia formation

**Fig. 3:**
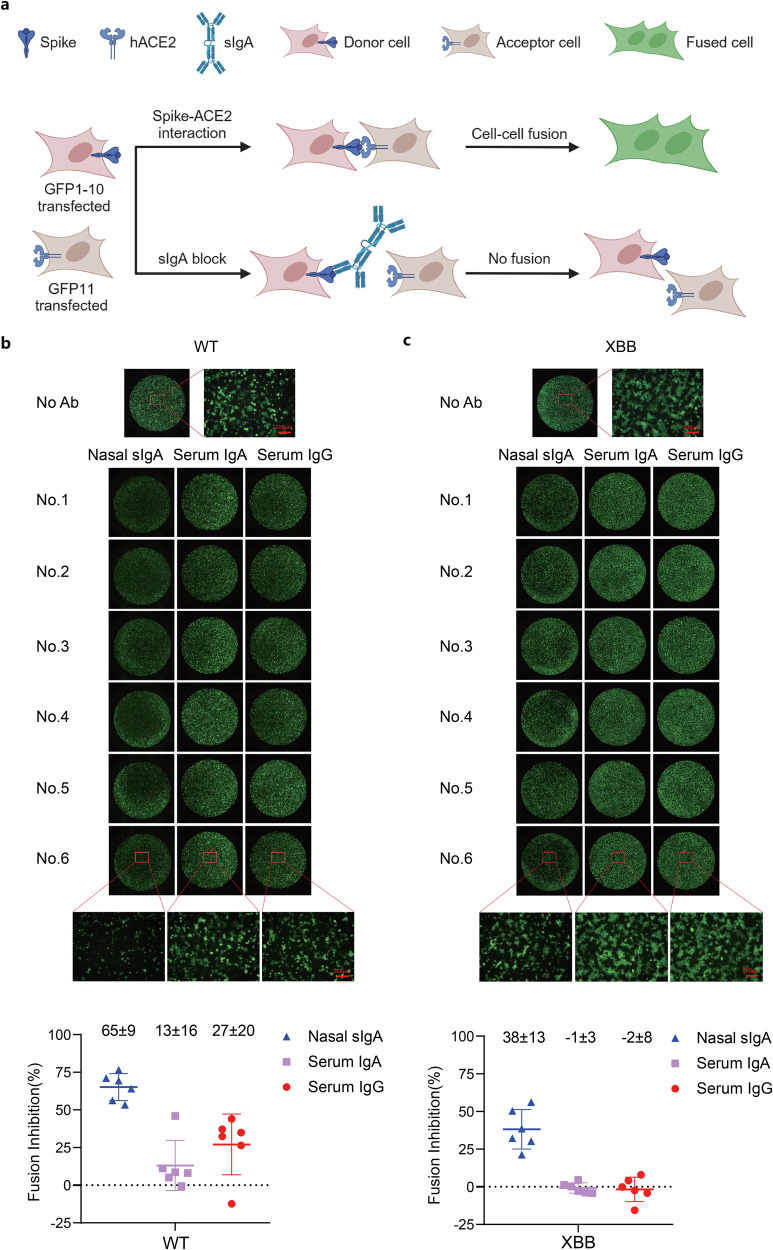
Comparison of nasal sIgA, serum IgG and IgA on inhibiting spike-mediated syncytia formation. **a**–**c** Inhibition of spike-mediated cell–cell fusion. The schematic is drawn using BioRender. 293T cells expressing WT- or XBB-spike and GFP_1-10_ were incubated with 150 μg of paired nasal sIgA, serum IgA, and serum IgG in 150 μL culture medium before co-culture with 293T cells expressing ACE2 and GFP_11_ in 96-well plates in a final volume of 200 μL. The formula for converting mass concentration to molar concentration is: Molar concentration (M) = Mass (mg)/(Volume (mL) × Molecular weight (g/mol or Da)). The molecular weight of dimeric IgA is ~400 kDa, which converts to a molar concentration of 2.5 μM. The molecular weight of serum monomeric IgG is approximately 150 kDa, converting to a molar concentration of 6.7 μM. Six hours later, images were captured with all-in-one fluorescence microscope BZ-X800LE, and fluorescence intensity was analyzed with supporting software to calculate the inhibition ratio. Shown are 6 pairs of nasal sIgA, serum IgA, and serum IgG samples collected simultaneously from 6 donors

The expression of a spike protein in SARS-CoV-2-infected cells can cause cell–cell fusion or syncytial formation to facilitate viral colonization and cell-to-cell transmission between epithelial cells in the upper respiratory tract.^16^ We compared the ability of paired serum IgA, serum IgG, and nasal sIgA in blocking spike-mediated syncytia formation. An assay using spike-expressing HEK293T cells co-cultured with hACE2-expressing HEK293T cells was used in which the green fluorescence could be detected if spike-mediated cell–cell fusion occurs (Fig. 3a). Serum IgG and IgA could neutralize WT (IC_50_ = 108 nM and 314 nM) but were less potent than sIgA (IC_50_ = 6 nM). At 1 mg/mL concentration, IgG and IgA could inhibit WT spike-mediated cell–cell fusion (27 ± 20% and 13 ± 16%) but were less potent than sIgA (65 ± 9%). However, IgG and IgA failed to neutralize XBB at 1 mg/mL concentration and could not inhibit XBB spike-mediated cell–cell fusion. Nasal sIgA (IC_50_ = 35 nM) still possessed potent neutralization against XBB and could inhibit XBB spike-mediated cell–cell fusion (38 ± 13%) (Fig. 3b, c). The ability of nasal sIgA to broadly inhibit spike-mediated syncytial formation is of significant implication in blocking intercellular transmission in the mucosal epithelium and preventing viruses across the mucosal barrier.

### Nasal sIgA is superior to serum IgA and IgG in protecting mice from intranasal XBB challenge

**Fig. 4:**
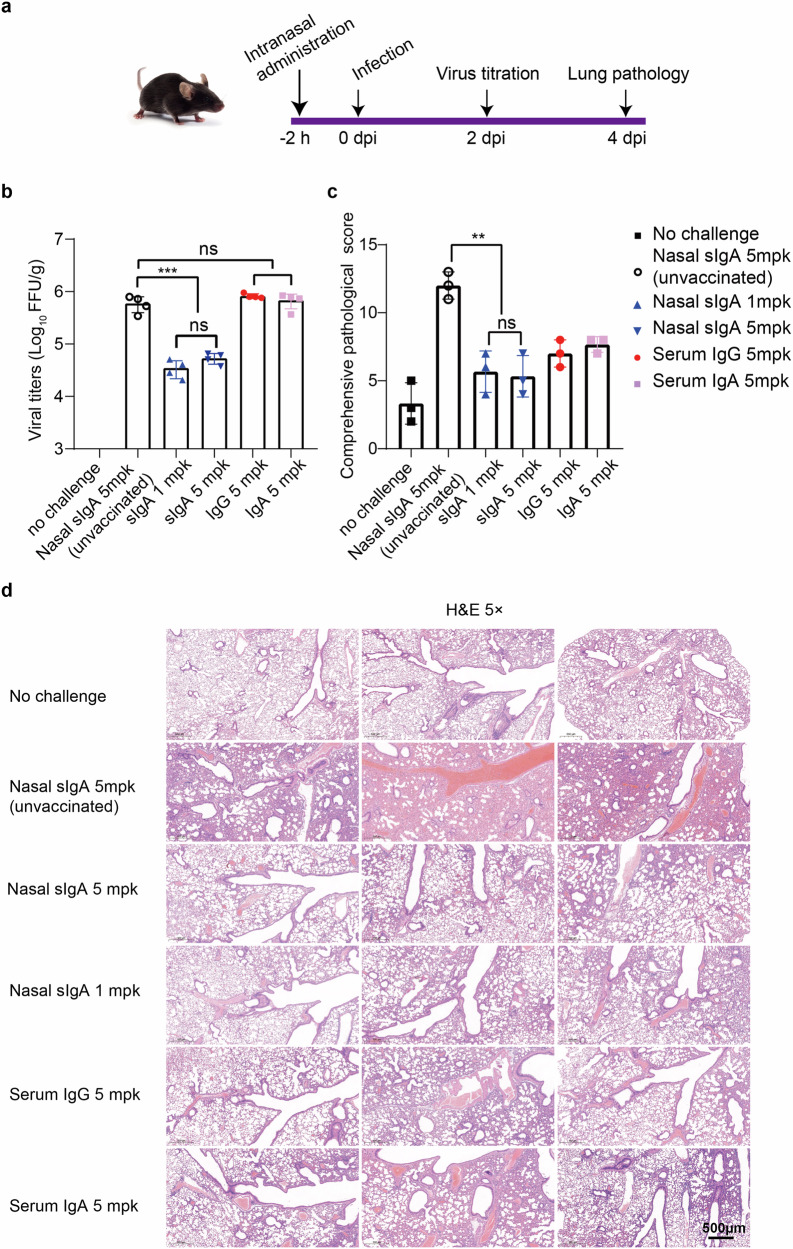
Comparison of nasal sIgA, serum IgG, and IgA on protecting mice against XBB challenge. **a** Mouse challenge study. The mouse was drawn using BioRender. Female 7-week-old K18-hACE2 transgenic mice received intranasal instillation of 1 or 5 mpk sIgA, or 5 mpk IgG or 5 mpk IgA pooled from 8 intranasal vaccinees. Mice that received intranasal instillation of 5 mpk sIgA from unvaccinated people were used as controls. Two hours later, mice were intranasally challenged with a bolus of Omicron XBB.1.1 (GDPCC-2.01503) at 50,000 FFU. Two days later, mice were sacrificed and the lungs were collected to measure infectious virus titers using fluorescence focus assay. Four days later, mice were sacrificed, and the lungs were collected for H&E (hematoxylin and eosin) staining. **b** Infectious virus titers in the lungs were measured by fluorescence focus assay. Statistical significance compared to the unvaccinated sIgA group was determined using ordinary one-way ANOVA (multiple comparisons), ^∗∗∗^*p* < 0.001; ns, not significant. **c** Comprehensive pathological scores of the lungs. Lung pathology scores were determined based on the following four parameters: alveolar edema/flooding, alveolar septum thickening and consolidation, bronchial infiltrates, and perivascular infiltrates. Statistical significance compared to the unvaccinated sIgA group was determined using an unpaired t test, ^∗∗^*p* < 0.01; ns, not significant. **d** Histopathological analysis of lung tissues. H&E staining of lung sections harvested at 4 days post-infection (dpi) or from mock-infected mice. Images are shown at 5× magnification. Representative images were obtained from 3 mice per group

The protective effects of serum IgA, IgG, and nasal sIgA were compared in a K18-hACE2 mouse model. We first assessed the pharmacokinetics of human nasal sIgA and serum IgG in the nasal cavity and lung of mice after a single intranasal instillation. At 1 h after intranasal instillation of 20 μg human sIgA or IgG, sIgA or IgG was present mainly in the lung (98%). At 48 h after intranasal instillation, sIgA and IgG in the lung were 4.03 μg/mL and 3.35 μg/mL, respectively. At 96 hours, there were no detectable sIgA and IgG in the lung and nasal cavity. We estimated that the half-life of human nasal sIgA and serum IgG in the lung of mice was 23.10 h and 18.95 h in mice (Supplementary Fig. 3). Next, purified sIgA, IgG, and IgA were pooled in equal portions from eight donors for in vivo comparison. Mice were divided into five groups, each containing seven mice: (1) nasal sIgA 1 mg/kg body weight (mpk), (2) nasal sIgA 5 mpk, (3) serum IgG 5 mpk, (4) serum IgA 5 mpk from intranasal vaccines, and (5) The control group received nasal sIgA 5 mpk from people without intranasal vaccination. After the instillation of these immunoglobulins into the nostrils, animals were challenged with 50,000 focus forming unit (FFU) Omicron XBB.1.1 via intranasal instillation. The lungs were collected for titration of live viruses and histopathological analysis (Fig. 4a). Compared with the control group, mice that received either 1 or 5 mpk of sIgA from vaccinees showed a significant and comparable reduction in viral load, suggesting 1 mpk is sufficient to exert the protective effect. In contrast, neither IgG nor IgA at 5 mpk significantly reduced the viral load in the lungs (Fig. 4b). Nasal sIgA from intranasal vaccinees, either 1 or 5 mpk sIgA, significantly decreased edema, infiltrates, and vascular damage than the control group. Serum IgG and IgA also showed modest alleviation compared to the control group, but edema, infiltrates, and vascular damage were visible (Fig. 4c, d, Supplementary Fig. 4). In addition, the expression of SARS-CoV-2 nucleocapsid protein was significantly lower in the lungs of the sIgA group compared with that in the IgG and IgA groups (Supplementary Fig. 5). These findings demonstrated that nasal sIgA, upon passively transferred into mouse nostrils, is more effective than serum IgG and IgA in protecting mice challenged with a large bolus of XBB viruses.

### sIgA is the dominant immunoglobulin in the nasal passage, and the production is robust

**Fig. 5:**
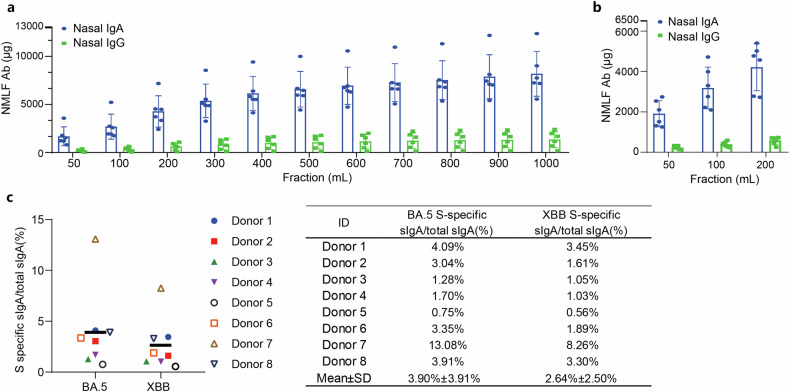
Estimated quantification of total sIgA and spike-specific sIgA in the nasal passage. **a** The amount of total nasal sIgA and IgG in the nasal passages. 1000 mL nasal lavage fluids were collected from each of the six donors using a nasal irrigator containing 1000 mL saline. IgA and IgG protein concentrations in each 100 mL fraction were measured using purified IgA and IgG as standard substances. Total protein concentration was determined using the bicinchoninic acid (BCA) method. Data are shown as mean ± SD (*n* = 6). **b** Production of nasal IgA and IgG in NMLFs after thorough washing. Eight hours after nasal washing with 1000 mL saline, 200 mL nasal lavage fluids were collected from each of the six donors using a nasal irrigator. The amount of IgA, IgG, and total protein were determined. Data are shown as mean ± SD (*n* = 6). **c** Estimation of the proportion of spike-specific sIgA in total nasal sIgA. BA.5 spike-specific and XBB spike-specific sIgA in each sample were measured by ELISA using mAb LC719-1 sIgA1 as a reference standard. The percentage of spike-specific sIgA was calculated based on the total nasal sIgA purified from each donor. Data are shown as mean ± SD (*n* = 8), and individual data are presented

To determine if the presence of sIgA in the nasal passage is sufficient to exert a protective effect, we estimated the amount of total sIgA and spike-specific sIgA in the nasal passage. We washed each donor’s nose with 1000 mL saline and used purified IgA and IgG as references for ELISA quantification (Fig. 5a, Supplementary Table 4). There was an average of 8.18 mg sIgA and 1.35 mg IgG, accounting for 39.4% and 6.5% of 20.78 mg total protein in 1000 mL nasal lavage fluids. The first 200 mL wash contained 4.28 mg sIgA and 0.61 mg IgG, accounting for 52% and 45% of total sIgA and IgG, respectively. In contrast, the last 200 mL wash contained 5% or less sIgA and IgG (Fig. 5a, Supplementary Table 4). Eight hours after nasal wash, we rewashed the noses with 200 mL saline and found 4.21 mg sIgA and 0.58 mg IgG in the 200 mL nasal lavage fluid, comparable to the amount from the first nasal wash (Fig. 5b, Supplementary Table 4). The nasal passage typically contains 10–20 mL fluids. Therefore, the concentration of total sIgA is 410–820 μg/mL or 1.0–2.0 μM, while the concentration of total IgG is 67.5–135.0 μg/mL or 0.45–0.90 μM in the nasal passage.

Next, we attempted to estimate the percentage of spike-specific sIgA in the total sIgA. LC719-1 is a monoclonal antibody (mAb) with comparable binding activities to all spikes we cloned from the nasal mucosa. Using LC719-1 sIgA mAb as a reference, we found that there was an average of 3.90% (range 0.75–13.08%) BA.5 spike-specific sIgA or 2.64% (range 0.56–8.26%) XBB spike-specific sIgA in total sIgA for NMLFs collected at 3–6 weeks after intranasal vaccination (Fig. 5c).

## Discussion

Our study provides evidence that intranasal vaccination using NB2155, an Ad5-vectored Omicron BA.1 vaccine, in individuals who previously received intramuscular injected ancestral vaccines can elicit potent and broadly neutralizing sIgA in the nasal passage against all variants tested so far. One intriguing finding was that although serum IgG and IgA were escaped by BQ.1.1, XBB, and EG.5, nasal sIgA maintained strong neutralizing potency against these later Omicron subvariants. This finding supported the assessment of nasal sIgA as an essential correlate of protection against infection. An intranasal vaccine that can induce broadly neutralizing nasal sIgA is more resilient to immune evasion than intramuscularly injected vaccines that induce serum antibodies. Therefore, developing variant-proof intranasal vaccines is possible, and they may not require frequent updates following the ever-changing circulating variants.

An earlier study demonstrated that converting mIgA mAbs to dIgA isoforms could enhance neutralizing potency against ancestral strains.^17^ Another paper reported that mAb Cv2.1169, isolated from circulating IgA memory B cells, its dIgA form is over 25-fold stronger than mIgA in neutralizing BA.1 and BA.2.^18^ Our study further demonstrated that sIgA from nasal mucosa, in the context of polyclonal and multimeric forms, could be over 2–3 logs stronger than serum IgA and IgG in neutralizing late Omicron subvariants, including XBB. Our findings that nasal sIgA can also potently neutralize pre-Omicron strains demonstrated that memory B cells elicited by earlier vaccination of inactivated SARS-CoV-2 vaccine contribute to the generation of cross-reactive mucosal sIgA after intranasal vaccination. In addition to the increased avidity conferred by the IgG to IgA isotype switching and the formation of dimeric and multimeric sIgA, whether the B cells producing sIgA in the nasal mucosa have further somatic hypermutation is unknown and should be studied in the future. However, obtaining B cells from human nasal mucosa can be difficult. Future studies may apply mass spectrometry or other new technologies, such as single-cell transcriptomic and proteomic technology, to compare the antibody sequences of serum IgA and IgG, and nasal sIgA.

Our study demonstrated that sIgA is the most critical and dominant immunoglobulin in the upper respiratory tract, providing the frontier defense. The daily production of IgA in humans is more significant than all other immunoglobulins combined.^19^ We found at least 8.2 mg of sIgA in the nasal passage, and the production is robust and can be replenished in a few hours after complete depletion. This finding can be meaningful for people who play water sports, such as swimming, who may temporarily lose sIgA in the nasal passage. The concentration of total sIgA in the nasal passage is 1–2 μM, which is much higher than the IC_50_ of nasal sIgA for neutralizing XBB (35 nM) and the other Omicron subvariants. In contrast, the concentration of 450 nM IgG in the nasal passage is insufficient to neutralize XBB and other subvariants. The finding that nasal sIgA, but not serum IgG or IgA, can effectively block WT and XBB spike-mediated syncytia formation has important implications. The viral infection starts in multiciliate cells and forms syncytia with basal cells. The viruses are released into the apical lumen and contribute to transmission.^20^ Therefore, the presence of sIgA in nasal and upper respiratory mucosa can play a critical role in preventing colonization and transmission. In addition to directly neutralizing the virus and blocking spike-mediated cell–cell fusion, polyclonal sIgA may have other mechanisms to hinder infection, such as antibody-dependent cell-mediated cytotoxicity (ADCC), immune exclusion, and intracellular neutralization,^21^ which should be investigated in future studies.

We reported in 2020 that a single intranasal vaccination using Ad5-S-nb2 (Ad5 carrying WT spike) conferred sterilizing-like protection against challenges in rhesus macaques.^14^ Our result differed from that of another study using intranasal ChAdOx1 nCoV-19,^22^ which only induced a weak elevation of spike-specific sIgA. ChAdOx1 is a simian-derived adenovirus (SAdY25) isolated from fecal samples and may not be the best vector for the respiratory tract. We believe that the selection of the virus, the design of the vector, the difference of adenovirus serotypes or species origin (which may have different tissue tropism), antigen selection (such as spike or RBD), and its gene optimization, formulation, and the methods of intranasal administration could all affect the efficacy in inducing mucosal sIgA and therefore protection efficacy.

Unlike intranasal vaccination, which results in persistent secretion of spike-specific sIgA in the upper respiratory tract, after a single intranasal instillation, the level of sIgA in the nasal cavity and lung decreases over time. Therefore, the mouse challenge model we used in this study aimed to compare the relative protection effectiveness of serum IgA and IgG with nasal sIgA. Of note, the 20 μg sIgA or IgG or IgA per mouse were polyclonal antibodies, and only a tiny proportion were spike-specific. Therefore, the actual dosage of spike-specific antibodies we used in the mouse challenge study was much lower than those using neutralizing mAbs, in which up to 300 μg per mouse was used in their mouse challenge study.^23,24^ Taken together, developing an easy and convenient approach to detecting spike-specific sIgA in the nasal passage and boosting mucosal immunity via intranasal vaccination will provide better and more precise prevention against future infections.

## Materials and methods

### Ethics statement and human subjects

In this study, written informed consent was obtained from all participants before enrollment. Nasal lavage fluid and serum samples were obtained from intranasal Ad5-S-Omicron vaccinated subjects. Detailed demographic and vaccine regimen information of the subjects in this study cohort were summarized in Supplementary Table 1. The study was carried out with the approval of the Medical Ethics Committee of the First Affiliated Hospital (No. 2022173) and Guangzhou Eighth People’s Hospital (No. 202303240), Guangzhou Medical University.

### Intranasal vaccination and sample collection in subjects

According to manufacturer’s instructions, intranasal vaccination in subjects was carried out. Briefly, Ad5-S-Omicron vaccine (2 × 10^10^ viral particles (vp) in 0.2 mL) was aspirated using the syringe. And then attached the syringe to the human nasal spray device. The spray device was affixed to the subject’s nostril and quickly atomized and delivers the vaccine into the subject’s nose. Repeat this vaccination operation to deliver the vaccine to the other nostril. Sera from subjects at different time points were obtained and heat-inactivated. NMLFs were collected either using a nasal swab to elute samples into 1.0 mL normal saline for immunogenicity study or using the human nasal irrigation device containing 200–1000 mL normal saline for nasal antibody purification. Protein levels in the NMLF were determined by a bicinchoninic acid (BCA) assay kit (Thermo Scientific, USA).

### Assay for cell‒cell fusion and inhibition

The split-GFP system was used for cell‒cell fusion assay (Addgene, USA). 1 × 10^6^ HEK293T-ACE2 or HEK293T cells per well were seeded into 6 well cell culture plates. The next day, HEK293T cells were transiently co-transfected using 1 μg pCDNA3.1-S-WT or pCDNA3.1-S-XBB and 2 μg pQCXIP-GFP_1-10_ using EZ Trans (Life-iLab, China), while HEK293T-ACE2 cells were transfected with pQCXIP-BSR-GFP_11_. Twenty-four hours after transfection, HEK293T-S-GFP1-10 cells and HEK293T-ACE2-GFP11 cells were digested and co-cultured at a density of 1 × 10^5^ cells/well (96-well plates) in Dulbecco’s modified eagle medium (DMEM) supplemented with 2% fetal bovine serum (FBS) for 8 h. Fluorescence images were recorded using an all-in-one fluorescence microscope BZ-X800LE (Keyence, Japan). S-mediated cell‒cell fusion was determined by observing multinucleated syncytium formation and green fluorescence occurrence. To measure the inhibition of cell‒cell fusion, purified sIgA, IgG, or IgA was prepared in culture medium and added into the cell culture well (96-well plates) at a concentration of 150 μg/150 μL. 1 × 10^5^ HEK293T-S-GFP1-10 cells per well were added into the wells and incubated for 1 h. 1 × 10^5^ HEK293T-ACE2-GFP11 cells per well were then added into the wells and incubated for another 6 h. The integration fluorescence intensity (IFI) of all cells in each well was measured by all-in-one fluorescence microscope BZ-X800LE (Keyence, Japan) to evaluate fusogenicity of spike protein. The antibody inhibition rate was calculated as follows: inhibition% = 1 − (IFI_antibody_ − IFI_blank_)/(IFI_control_ − IFI_blank_).

### Fractionation of nasal IgA in nasal wash samples using size exclusion chromatography

First, the Superose™ 6 increase 10/300 GL gel filtration column (Cytiva, Sweden) on the FPLC AKTA Chromatography System was calibrated using the Gel Filtration HMW Calibration Kit (Cytiva, Sweden) and IgG at room temperature. After column equilibration, purified nasal IgA was placed into the column and the flow rate was set at 0.5 mL/min. The multimers, dimers, and monomers of nasal IgA were separated according to their molecule weights. The fractions (1 mL each) were collected in phosphate puffer saline (PBS). These fractions were concentrated, and then evaluated using SDS-PAGE under non-reducing conditions. Coomassie blue staining and western blot analysis were then performed.

### Multiplex electrochemiluminescence assay

SARS-CoV-2 spike-specific IgA was measured in NLMFs or serum by using the V-PLEX SARS-CoV-2 Panel 29 (IgA) Kit (MSD, USA). Briefly, NLMF or serum samples were diluted 1:100- or 1:3200-fold, respectively, using a diluent 100 solution. The reference standard serum was diluted in a 4-fold dilution series with an initial 200-fold dilution to calculate antibody concentrations and assign arbitrary units (AU/mL). Assay plates were treated with blocking solution A for 30 min. Diluted NLMF or serum samples were added into the plate and then anti-human IgA antibody (SULFO-TAG) was added. BSA was added into each well as the negative control. The MSD GOLD Read buffer was added and analyzed immediately by using the MSD detection system (MESO SECTOR S 600MM). Protein levels in the NMLF samples were determined by a BCA assay kit (Thermo Scientific, USA) and the antibody titer was normalized.

### Neutralization assay based on authentic SARS-CoV-2

Vero E6 cells (ATCC, USA) were cultured in DMEM medium supplemented with 10% FBS, 100 μg/mL streptomycin, and 100 IU/mL penicillin. SARS-CoV-2 Omicron BA.1.1 variant (IQTC-IM21Y6017) and XBB.1.1 variant (GDPCC-2.01503) was propagated in Vero E6 cells. Viral titers were titrated by using Vero E6 cells. The cytopathic effect (CPE) score was performed 3 days after infection, and 50% Tissue culture infectious dose (TCID_50_) was calculated by using the Reed-Muench formula. All SARS-CoV-2-related infection procedures were carried out at biosafety level-3 (BSL-3) laboratory at the Guangzhou Customs Inspection and Quarantine Technology Center (IQTC).

Different dilutions of the antibodies were incubated with Omicron BA.1.1 or XBB.1.1 (multiplicity of infection (MOI) = 0.01). After 1 h of incubation, 135 μL mixtures were added into Vero E6 cells (2 × 10^4^/well) in a 96-well plate. Three days after infection, the CPE score was performed by using a Celigo^TM^ Image Cytometer. Half-maximal neutralizing titer and antibody inhibition were calculated.

### Vesicular stomatitis virus (VSV) pseudoviruses-based neutralization assay

Pseudoviral neutralization assays against serum IgA, serum IgG, and nasal sIgA were carried out as previously described.^25^ Briefly, purified nasal sIgA or serum IgG and IgA were measured at 37.5 or 337.5 μg/mL, respectively, with six consecutive 1:3 dilutions in DMEM. NMLFs or Serum were tested at an initial dilution of 1:10 (NMLFs) or 1:30 (serum) and six consecutive 1:3 dilutions in DMEM. The diluted samples were incubated with pseudovirus (650 TCID_50_) in the 96-well plate (JETBIOFIL, China). After 1 h, a mixture of 100 μL from each well was then added to the 96-well plate containing 20,000 Huh-7 cells. After incubation for 24 h, the reduction in relative luminescence unit (RLU) compared to positive control (virus-infected cells) was assessed by using the Bio-LiteTM luciferase assay system (Vazyme, China). 50% neutralization titer (NT_50_) and 50% inhibitory concentration (IC_50_) were calculated by using Reed-Muench method and four-parameter nonlinear regression, respectively.

### Purification of serum IgA, serum IgG, and nasal sIgA

The serum samples were diluted 1:10 in PBS. IgG was purified by using Protein G Sepharose^TM^ 4 Fast Flow (Cytiva, Sweden). IgG was eluted with 10 mL of glycine (0.1 M, pH 3) and neutralized with Tris-HCl (1.5 M, pH 8.8). NLMFs were concentrated by using ultracentrifugation filters with a molecular weight cutoff of 100 kDa (Millipore, USA). Nasal and serum IgA were purified by using peptide M/agarose (InvivoGen, France). Purified serum IgG, IgA, and nasal sIgA were concentrated by using ultracentrifugation filters with a molecular weight cutoff of 100 kDa (Millipore, USA). The absorbance at 280 nm was measured using a Nanodrop instrument (Thermo Fisher Scientific, USA) to determine the concentration of purified antibodies. The purified antibodies were used for subsequent binding and neutralization analyses.

### Sedimentation velocity-analytical ultracentrifugation (SV-AUC)

SV-AUC were performed by using Optima AUC analytical-ultracentrifuge (Beckman Coulter, USA) at 12 °C and 35,000 rpm with absorbance measure of 280 nm. SEDFIT software was used to analyze the data, and the sedimentation coefficient distribution C (S) was obtained. Consider any settling coefficient values greater than 20 as instrument errors.

### SDS‒PAGE and Western blot analysis

Donor NMLFs were subjected to SDS-PAGE and analyzed after Coomassie blue staining. For western blot analysis, purified nasal sIgA, human serum, and serum IgG were used as references. Polyvinylidene fluoride (PVDF) membranes were then incubated with horseradish peroxidase (HRP)-conjugated mouse anti-human IgM antibody (SouthernBiotech, USA), goat anti-human IgE antibody (Invitrogen, USA), goat anti-human IgA heavy chain (HC) antibody (Abcam, UK), goat anti-human IgD heavy chain antibody (GeneTex, USA), and goat anti-human IgG Fc antibody (Abcam, UK). Bio-Rad imaging system was used to develop PVDF membrane by adding chemiluminescent HRP substrate (Millipore, USA).

### ELISA

0.2 μg spike protein per well (Sino Biological, China) for IgA or 0.05 μg spike protein per well for IgG were coated in 96-well plate overnight at 4 °C. The plates were blocked with 1×PBS supplemented with 5% skim milk for 2 h at 37 °C. Six serial 3-fold dilutions of purified antibodies at a starting concentration of 125 μg/mL (serum antibodies) or 37.5 μg/mL (nasal antibodies) were added into plate. After 2 h, the plates were incubated with HRP-conjugated goat anti-human IgA antibodies (Abcam, UK) or HRP-conjugated goat anti-human IgG H + L antibodies (Beyotime Biotechnology, China). After incubation for another 1 h at 37 °C, 3,3’,5,5’-tetramethylbenzidine (TMB) substrate (Millipore, USA) was added to the reaction and measured at 450 nm. Four-parameter nonlinear regression was used to determine 50% effective concentration (EC_50_) of antibodies.

### SARS-CoV-2 XBB.1 challenge study

The use of mice was approved by the Animal Ethics Committee of Guangzhou Customs Inspection and Quarantine Technology Center (No. 20230522). Eight-week-old female H11-K18-hACE2 Tg mice (T037657) were purchased from Gem Pharmaceutical. Mice were anesthetized with isoflurane and intranasally administered sIgA antibodies at 1 and 5 mpk and IgG, IgA, and sIgA isotypes at 5 mpk. Two hours after antibody administration, mice were challenged with 5 × 10^4^ FFU XBB.1, prediluted in 50 μL DMEM. Two days after infection, lung tissues were collected to measure viral titers using a fluorescence focus assay.

Four days after infection, lung tissues were collected for histopathological and immunohistochemistry (IHC) analyses. Histological staining was performed using the established protocols.^26^ Briefly, lung tissues were fixed, embedded, and cut into sections. For hematoxylin and eosin (H&E) staining, sections were stained with Gill’s H&E Y (Thermo Scientific, USA). Images were obtained by using a Pannoramic MIDI system (3DHISTECH, Hungary). For IHC assay, sections were mounted on Super-frost Plus Microscope slides, and stained with rabbit anti-SARS-CoV-2 N protein antibodies (Immunoway, USA) at 1:1600 dilution, then were detected with a secondary antibody. Finally, the slides were incubated with DAPI. Images were collected using a KFBIO Digital Pathology Slide Scanner (KFBIO, China).

Lung tissue pathology scoring criteria in this study was based on recent at K18-hACE2 transgenic mouse model of SARS-CoV-2 infection research.^27^ Briefly, the severity of pathological changes in lung tissue was analyzed by H&E staining. The pathological score includes: (a) alveolar edema, mucus, hemorrhage, and exudation; (b) alveolar septum thickening and consolidation; (c) peribronchiolar/bronchial inflammation; (d) perivascular inflammation. Score related to the severity (0, no pathological change; 1, pathological change in normal range; 2, mild pathological change; 3, moderate pathological change; and 4, severe pathological change).

### Pharmacokinetics of human nasal sIgA and serum IgG in the nasal cavity and lung

Six-week-old female Balb/c mice were anesthetized with isoflurane and intranasally administered nasal sIgA, serum IgG at 1 mpk (20 μg antibody/mouse). Nasal lavage fluid (NLF) and broncho-alveolar lavage fluid (BALF) were collected at 1, 8, 24, 48, and 96 h after antibody administration. An ELISA assay was used to determine sIgA or IgG concentrations in mouse BALF or NLF. Briefly, the 96-well ELISA plates were coated with 100 μl per well of rabbit anti-human IgA H&L (Abcam, UK) or goat anti-human IgG Fc (Sigma-Aldrich, USA). 100 μl serial dilutions of BALF or NLF were added to the wells and incubated at 37 °C for 2 h. HRP-conjugated goat anti-human IgA alpha chain (Abcam, UK) or HRP-conjugated goat anti-human IgG heavy chain (Invitrogen, USA) were used as detection antibodies. Human serum-derived IgG and nasal wash-derived sIgA are used as standard references. Exponential decay curve fitting was used to determine antibody half-life (GraphPad Prism).

### Statistical analyses

The EC_50_ values were determined by fitting the data to a four-parameter nonlinear regression using GraphPad Prism. The IC_50_ values were determined by Reed-Muench. Comparisons between groups were conducted using unpaired Students’ t-test (two-tailed). *p* < 0.05 was considered statistically significant. Images were assembled using Adobe Illustrator.

### Supplementary information

Sigtrans_Supplementary_Materials_SIGTRANS-12586R1

## Data Availability

All data generated or analyzed during this study are available from the corresponding authors upon reasonable request.
