# Supplementary material for: Intranasal adenovirus-vectored Omicron vaccine induced nasal immunoglobulin A has superior neutralizing potency than serum antibodies
Source: Signal Transduct Target Ther. 2024 Jul 22;9:190. doi: 10.1038/s41392-024-01906-0 (PMC11263566; doi:10.1038/s41392-024-01906-0)
Supplement: Supplementary file 1 — Sigtrans_Supplementary_Materials_SIGTRANS-12586R1 [file 41392_2024_1906_MOESM1_ESM.docx]

Supplementary Materials for

**Intranasal adenovirus-vectored Omicron vaccine induced nasal immunoglobulin A has superior neutralizing potency than serum antibodies**

Si Chen^#^, Zhengyuan Zhang^#^, Qian Wang^#^, Qi Yang^#^, Li Yin, Lishan Ning, Zhilong Chen, Jielin Tang, Weiqi Deng, Ping He, Hengchun Li, Linjing Shi, Yijun Deng, Zijian Liu, Hemeng Bu, Yaohui Zhu, Wenming Liu, Linbing Qu, Liqiang Feng, Xiaoli Xiong, Baoqing Sun, Nanshan Zhong, Feng Li, Pingchao Li^*^, Xinwen Chen^*^, Ling Chen^*^

^*^Correspondence to: chen_ling@gibh.ac.cn; chen_xinwen@gzlab.ac.cn; li_pingchao@gibh.ac.cn

**This PDF file includes:**

Figures. S1 to S5

Tables S1 to S4


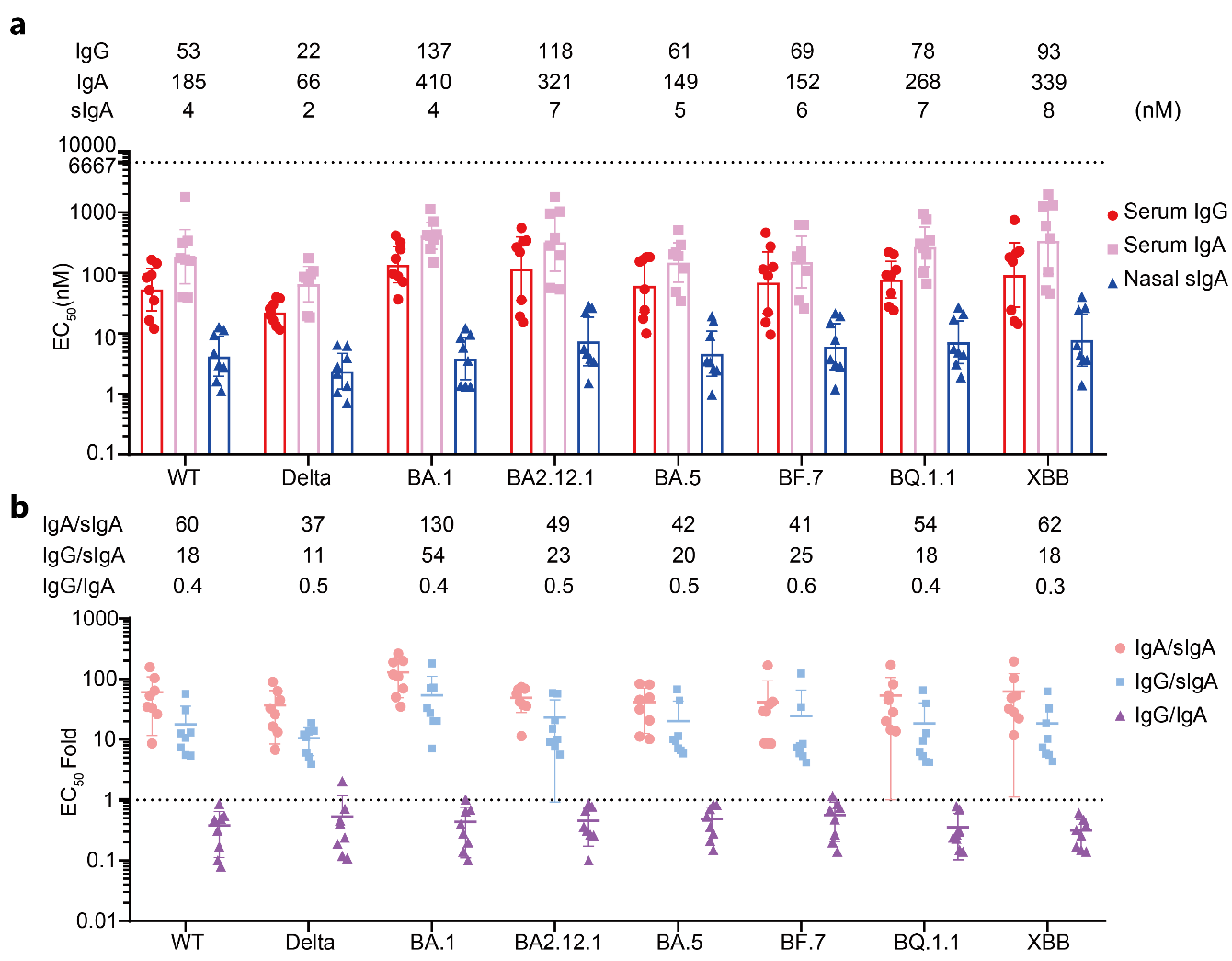


**Figure. S1. Binding activities of paired nasal sIgA, serum IgG, and serum IgA to spikes of pre-Omicron strains and Omicron subvariants.**


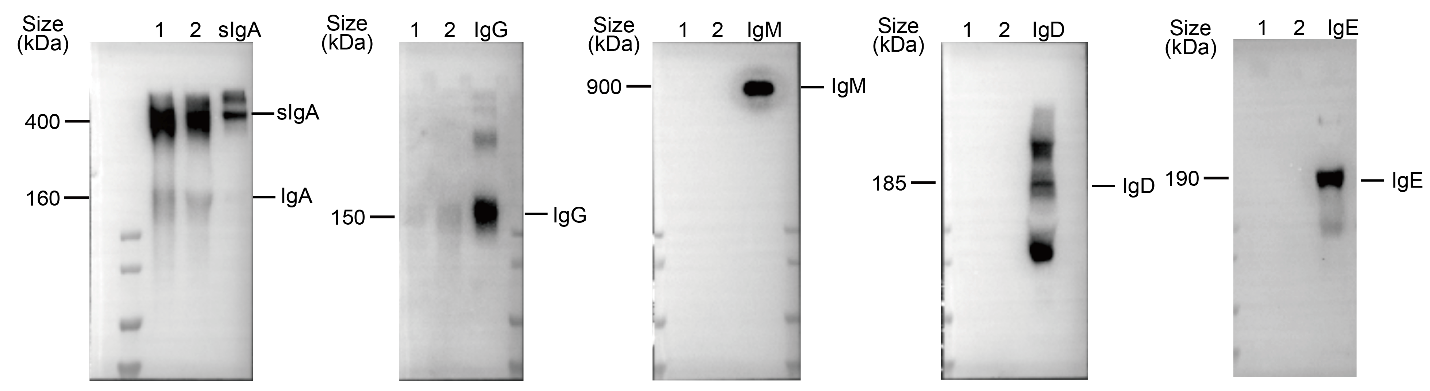


**Figure. S2. Western blot analysis of IgA, IgG, IgM, IgD, and IgE in nasal mucosal lining fluids (NMLFs).**


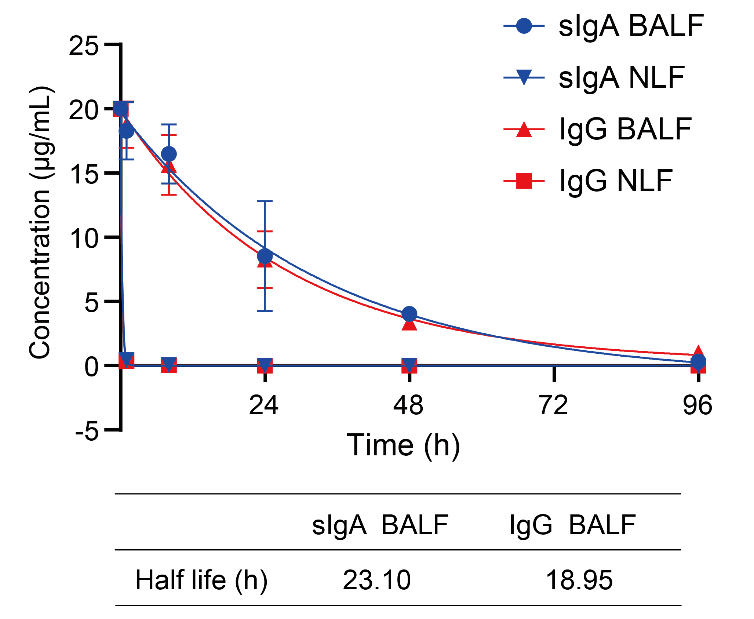


**Figure. S3. Pharmacokinetics of human nasal sIgA and serum IgG in the nasal cavity and lung.**


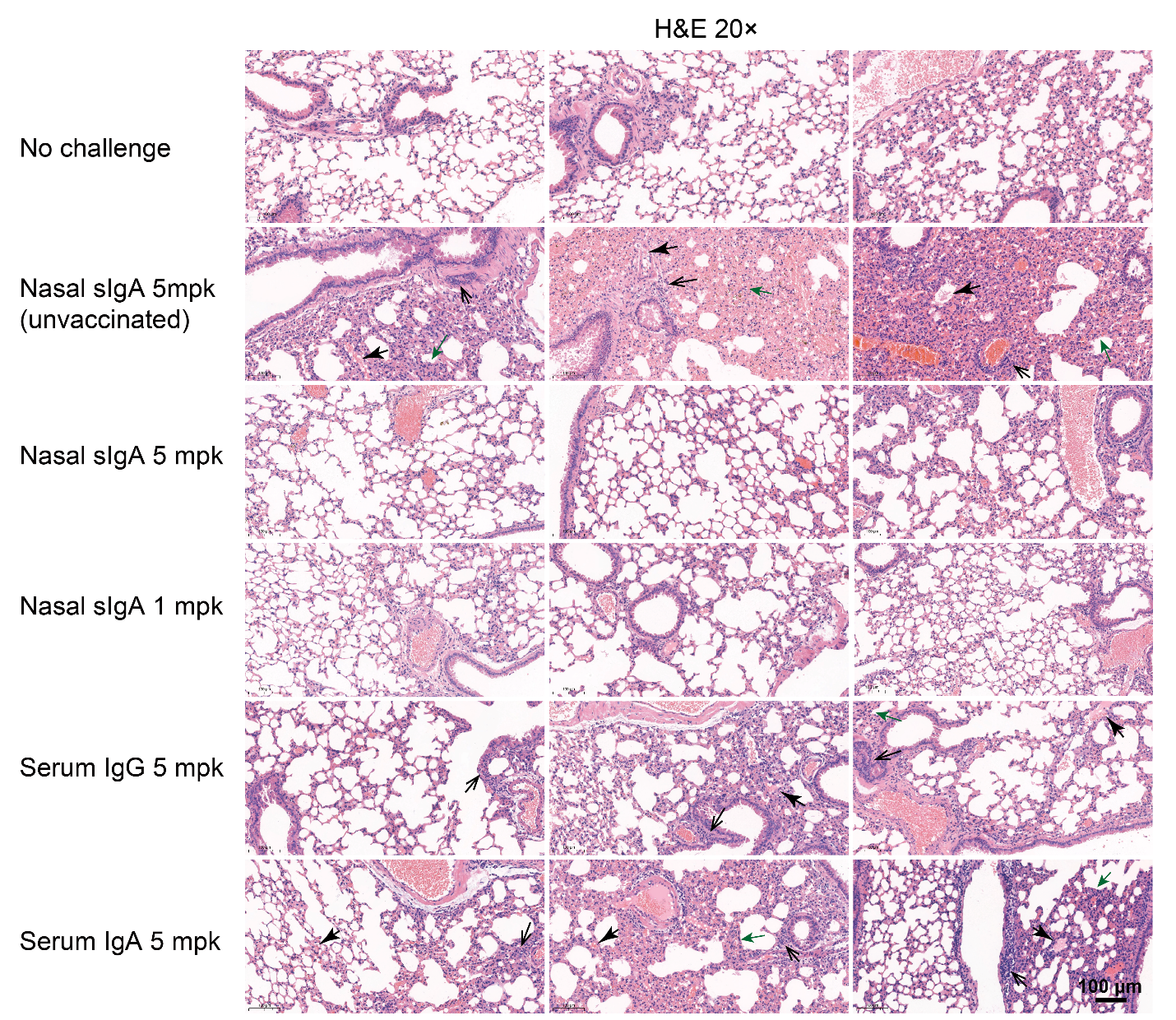


**Figure. S4. Histopathological analysis of lung tissues.**


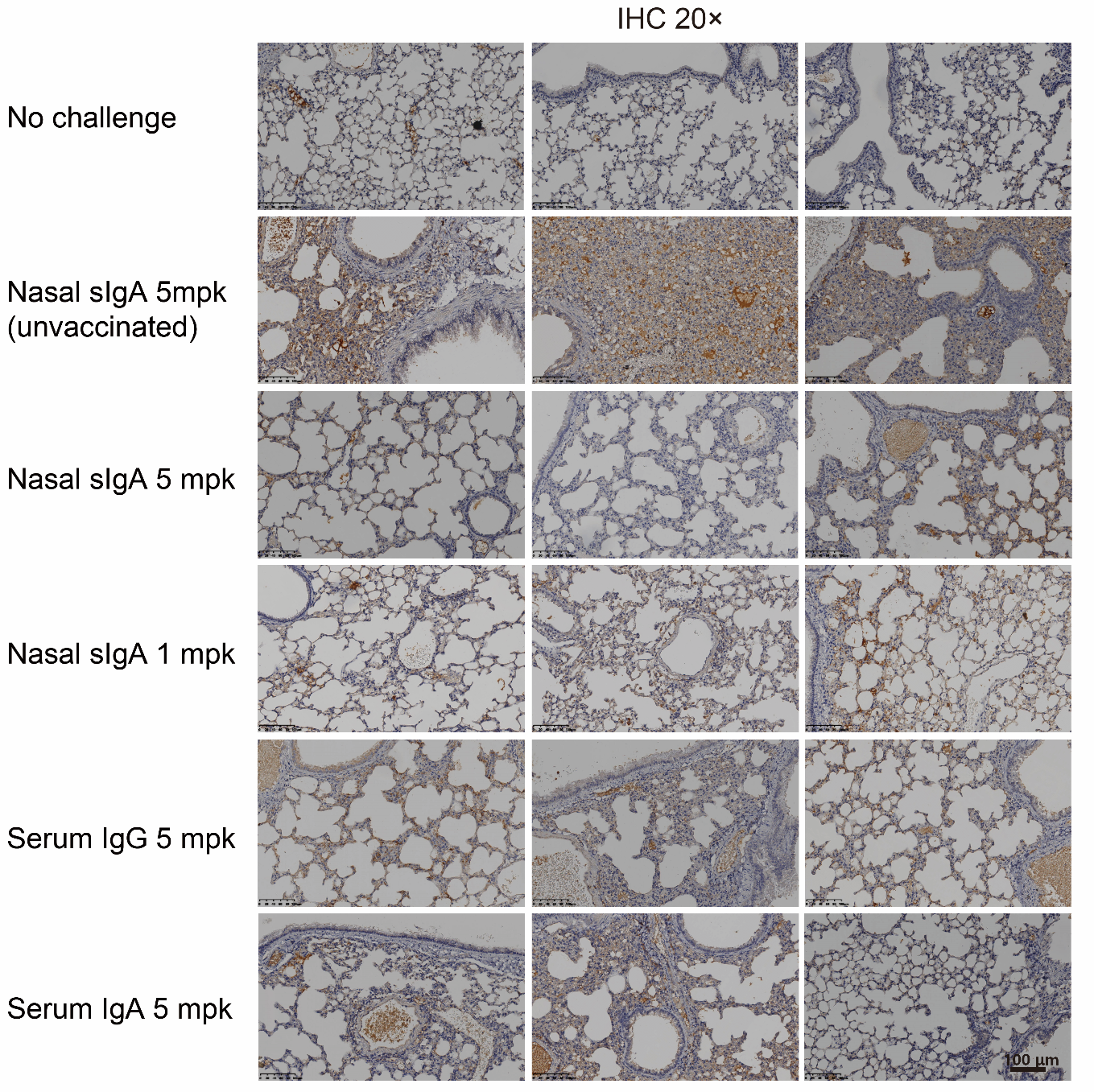


**Figure. S5. The staining for SARS-CoV-2 nucleocapsid protein using immunohistochemistry.**

| **Donor ID** | **Age**  **(year)** | **Gender**  **(F/M)** | **Doses of WT vaccine** | **Doses of**  **intranasal vaccine** | **Interval between WT vaccine and intranasal vaccine (month)** | **Sample collection time (days post intranasal vaccination)** |
| --- | --- | --- | --- | --- | --- | --- |
| **1** | **60** | **M** | **3** | **2** | **7** | **27** |
| **2** | **41** | **M** | **2** | **2** | **7** | **27** |
| **3** | **27** | **F** | **2** | **2** | **15** | **20** |
| **4** | **25** | **F** | **3** | **2** | **7** | **20** |
| **5** | **26** | **M** | **2** | **2** | **15** | **20** |
| **6** | **43** | **F** | **2** | **2** | **11** | **39** |
| **7** | **26** | **F** | **3** | **2** | **11** | **31** |
| **8** | **24** | **M** | **3** | **2** | **12** | **24** |

**Table S1. Demographics and vaccine regimens of the study cohort**

|  | **Serum IgG (nM)** | | | | | | | | **Geomean** | **95% CI** | |
| --- | --- | --- | --- | --- | --- | --- | --- | --- | --- | --- | --- |
|  | **1** | **2** | **3** | **4** | **5** | **6** | **7** | **8** |  | **Upper Limit** | **Lower Limit** |
| **WT** | 166.9 | 113.7 | 154.5 | 171.3 | 220.2 | 58.8 | 28.1 | 102.0 | 108.1 | 190.6 | 61.3 |
| **Delta** | 155.5 | 114.5 | 322.9 | 972.1 | 1424.9 | 84.3 | 27.3 | 177.8 | 206.1 | 606.9 | 70.0 |
| **BA.1** | 199.1 | 212.2 | 213.1 | 855.7 | 195.0 | 65.6 | 65.6 | 80.1 | 163.8 | 334.9 | 80.1 |
| **BA.5** | 865.2 | 512.6 | 421.5 | 1056.8 | 1138.1 | 228.2 | 122.6 | 212.6 | 437.3 | 870.4 | 219.7 |
| **BF.7** | 1440.0 | 847.4 | 551.6 | 794.2 | 885.8 | 272.7 | 156.6 | 138.1 | 479.4 | 992.7 | 231.6 |
| **BQ.1.1** | 6666.7 | 1813.3 | 1021.8 | 6666.7 | 6666.7 | 6666.7 | 6666.7 | 1001.8 | 3536.1 | 7463.3 | 1675.4 |
| **XBB** | 6666.7 | 6666.7 | 6666.7 | 6666.7 | 1966.5 | 6666.7 | 6666.7 | 2061.3 | 4942.1 | 7856.0 | 3109.0 |
| **XBB.1.5** | 6666.7 | 6666.7 | 6666.7 | 6666.7 | 1871.5 | 6666.7 | 6666.7 | 1188.1 | 4584.7 | 8257.5 | 2545.5 |
|  |  |  |  |  |  |  |  |  |  |  |  |
|  | **Serum IgA (nM)** | | | | | | | | **Geomean** | **95% CI** | |
|  | **1** | **2** | **3** | **4** | **5** | **6** | **7** | **8** |  | **Upper Limit** | **Lower Limit** |
| **WT** | 146.6 | 286.0 | 785.1 | 1441.2 | 652.7 | 61.8 | 121.6 | 405.0 | 313.9 | 762.6 | 129.2 |
| **Delta** | 102.6 | 198.4 | 726.8 | 831.5 | 837.9 | 47.0 | 152.7 | 1049.7 | 306.3 | 817.9 | 114.7 |
| **BA.1** | 143.9 | 215.0 | 403.6 | 978.5 | 285.5 | 30.2 | 112.1 | 531.9 | 223.7 | 547.5 | 91.4 |
| **BA.5** | 1097.1 | 630.9 | 716.1 | 968.5 | 367.7 | 45.3 | 370.5 | 1161.9 | 492.1 | 1196.1 | 202.5 |
| **BF.7** | 1560.4 | 639.3 | 681.1 | 1088.9 | 389.6 | 69.6 | 711.6 | 1063.1 | 592.4 | 1323.6 | 265.2 |
| **BQ.1.1** | 6666.7 | 6666.7 | 1043.1 | 1294.1 | 1077.6 | 652.4 | 6666.7 | 6666.7 | 2565.1 | 6563.5 | 762.2 |
| **XBB** | 6666.7 | 6666.7 | 6666.7 | 6666.7 | 2185.4 | 532.4 | 6666.7 | 6666.7 | 4228.2 | 9152.8 | 1953.2 |
| **XBB.1.5** | 6666.7 | 6666.7 | 6666.7 | 6666.7 | 1368.2 | 624.8 | 6666.7 | 1623.8 | 3409.9 | 7637.2 | 1522.5 |
|  |  |  |  |  |  |  |  |  |  |  |  |
|  | **Nasal sIgA (nM)** | | | | | | | | **Geomean** | **95% CI** | |
|  | **1** | **2** | **3** | **4** | **5** | **6** | **7** | **8** |  | **Upper Limit** | **Lower Limit** |
| **WT** | 2.6 | 2.9 | 38.6 | 15.2 | 29.4 | 4.7 | 1.5 | 3.5 | 6.5 | 17.9 | 2.3 |
| **Delta** | 4.0 | 4.2 | 25.0 | 21.3 | 26.5 | 0.4 | 1.2 | 2.5 | 4.7 | 17.4 | 1.3 |
| **BA.1** | 3.2 | 5.2 | 33.0 | 17.5 | 11.7 | 1.4 | 0.9 | 3.8 | 5.2 | 14.6 | 1.9 |
| **BA.5** | 16.8 | 13.7 | 33.0 | 20.2 | 11.0 | 0.5 | 2.2 | 3.6 | 7.1 | 23.1 | 2.2 |
| **BF.7** | 15.7 | 8.8 | 33.4 | 27.0 | 13.9 | 1.4 | 3.9 | 7.4 | 9.6 | 22.9 | 4.0 |
| **BQ.1.1** | 41.8 | 39.6 | 75.5 | 35.1 | 40.4 | 0.8 | 8.7 | 5.5 | 16.9 | 60.1 | 4.8 |
| **XBB** | 55.0 | 50.5 | 99.3 | 47.1 | 51.0 | 20.1 | 16.2 | 9.5 | 34.6 | 66.4 | 18.0 |
| **XBB.1.5** | 163.7 | 80.9 | 74.3 | 32.1 | 33.8 | 1.6 | 12.9 | 9.0 | 25.7 | 89.1 | 7.4 |

**Table S2. Pseudovirus neutralization IC_50_ values in each individual for serum IgG, IgA, and nasal sIgA against Omicron subvariants BA.1, BA.5, BF.7, BQ.1.1, XBB, XBB.1.5 and pre-Omicron WT and Delta**

|  | **IC_50_ Fold (Nasal sIgA/Serum IgA)** | | | | | | | | **Mean ± SD** |
| --- | --- | --- | --- | --- | --- | --- | --- | --- | --- |
|  | **1** | **2** | **3** | **4** | **5** | **6** | **7** | **8** |  |
| **WT** | 56.1 | 100.5 | 20.4 | 95.0 | 22.2 | 13.1 | 83.7 | 114.9 | 63.2±40.7 |
| **Delta** | 25.6 | 47.8 | 29.1 | 39.0 | 31.6 | 129.1 | 129.9 | 415.5 | 105.9±132.4 |
| **BA.1** | 44.4 | 41.5 | 12.2 | 55.9 | 24.4 | 21.8 | 122.2 | 139.2 | 57.7± 47.4 |
| **BA.5** | 65.2 | 45.9 | 21.7 | 48.1 | 33.3 | 96.6 | 169.6 | 325.5 | 100.7±102.2 |
| **BF.7** | 99.5 | 72.3 | 20.4 | 40.4 | 28.0 | 50.5 | 182.6 | 143.5 | 79.6± 58.2 |
| **BQ.1.1** | 159.5 | 168.5 | 13.8 | 36.9 | 26.7 | 813.7 | 763.3 | 1209.6 | 399.0± 461.4 |
| **XBB** | 121.1 | 131.9 | 67.2 | 141.6 | 42.9 | 26.5 | 412.4 | 704.2 | 206.0± 234.7 |
| **XBB.1.5** | 40.7 | 82.4 | 89.7 | 207.8 | 40.5 | 403.9 | 517.7 | 179.6 | 195.3± 177.1 |
|  |  |  |  |  |  |  |  |  |  |
|  | **IC_50_ Fold (Nasal sIgA/Serum IgG)** | | | | | | | | **Mean ± SD** |
|  | **1** | **2** | **3** | **4** | **5** | **6** | **7** | **8** |  |
| **WT** | 63.9 | 40.0 | 4.0 | 11.3 | 7.5 | 12.5 | 19.4 | 29.0 | 23.4±20.2 |
| **Delta** | 38.8 | 27.6 | 12.9 | 45.6 | 53.7 | 231.8 | 23.2 | 70.4 | 63.0±70.6 |
| **BA.1** | 61.5 | 40.9 | 6.5 | 48.9 | 16.7 | 47.4 | 71.5 | 21.0 | 39.3±22.7 |
| **BA.5** | 51.5 | 37.3 | 12.8 | 52.5 | 103.1 | 486.7 | 56.1 | 59.5 | 107.4±155.3 |
| **BF.7** | 91.8 | 95.8 | 16.5 | 29.5 | 63.7 | 197.9 | 40.2 | 18.6 | 69.2±60.4 |
| **BQ.1.1** | 159.5 | 45.8 | 13.5 | 190.2 | 165.1 | 8315.1 | 763.3 | 181.8 | 1229.3±2872.50 |
| **XBB** | 121.1 | 131.9 | 67.2 | 141.6 | 38.6 | 331.6 | 412.4 | 217.7 | 182.8±130.0 |
| **XBB.1.5** | 40.7 | 82.4 | 89.7 | 207.8 | 55.4 | 4310.1 | 517.7 | 131.4 | 679.4±1475.1 |
|  |  |  |  |  |  |  |  |  |  |
|  | **IC_50_ Fold (Serum IgA/Serum IgG)** | | | | | | | | **Mean ± SD** |
|  | **1** | **2** | **3** | **4** | **5** | **6** | **7** | **8** |  |
| **WT** | 1.1 | 0.4 | 0.2 | 0.1 | 0.3 | 1.0 | 0.2 | 0.3 | 0.5±0.4 |
| **Delta** | 1.5 | 0.6 | 0.4 | 1.2 | 1.7 | 1.8 | 0.2 | 0.2 | 0.9±0.7 |
| **BA.1** | 1.4 | 1.0 | 0.5 | 0.9 | 0.7 | 2.2 | 0.6 | 0.2 | 0.9±0.6 |
| **BA.5** | 0.8 | 0.8 | 0.6 | 1.1 | 3.1 | 5.0 | 0.3 | 0.2 | 1.5±1.7 |
| **BF.7** | 0.9 | 1.3 | 0.8 | 0.7 | 2.3 | 3.9 | 0.2 | 0.1 | 1.3±1.3 |
| **BQ.1.1** | 1.0 | 0.3 | 1.0 | 5.2 | 6.2 | 10.2 | 1.0 | 0.2 | 3.1±3.7 |
| **XBB** | 1.0 | 1.0 | 1.0 | 1.0 | 0.9 | 12.5 | 1.0 | 0.3 | 2.3±4.1 |
| **XBB.1.5** | 1.0 | 1.0 | 1.0 | 1.0 | 1.4 | 10.7 | 1.0 | 0.7 | 2.2±3.4 |

**Table S3. The ratio of IC_50_ between paired sIgA/IgA, sIgA/IgG, and IgA/IgG for each individual**

|  | **In total 1000 mL** | **In 1st 100 mL** | **In 1st 200 mL** | **In last 200 mL** | **Post-8h in 1^st^ 100 mL** | **Post-8h in 1^st^ 200 mL** |
| --- | --- | --- | --- | --- | --- | --- |
| **IgA (μg)** | 8182.19±  2328.35 | 2689.74±  1300.87 | 4277.16±  1624.51 | 416.09±  106.46 | 3178.44±  1029.99 | 4207.90±  1165.22 |
| **(IgA/total IgA) ×100%** |  | 32.9% | 52.3% | 5.1% | 38.8% | 51.4% |
| **IgG (μg)** | 1348.88±  829.70 | 357.53±  219.36 | 610.25±  377.63 | 58.15±  27.2 | 394.50±  136.23 | 580.16±  192.85 |
| **(IgG/total IgG) ×100%** |  | 26.5% | 45.2% | 4.3% | 29.2% | 43.0% |
| **Total protein (μg)** | 20784.88±  2719.65 | 3952.75±  1559.03 | 6845.04±  2012.62 | 2813.14±  910.23 | 4474.56±  688.95 | 6905.54±  862.16 |
| **(IgA/total protein) ×100%** | 39.37% | 68.05% | 62.49% | 14.79% | 71.03% | 60.94% |
| **(IgG/total protein) ×100%** | 6.49% | 9.05% | 8.92% | 2.07% | 8.82% | 8.40% |

**Table S4. Measurement of sIgA and IgG in NMLFs**
